# Glycoproteomics and Its Role in Understanding Bacterial O‐Linked Glycosylation

**DOI:** 10.1002/pmic.202400256

**Published:** 2025-04-21

**Authors:** Kristian I. Karlic, Hamza Tahir, Nichollas E. Scott

**Affiliations:** ^1^ Department of Microbiology and Immunology University of Melbourne at the Peter Doherty Institute for Infection and Immunity Melbourne Australia

**Keywords:** bacteria, glycoproteomics, O‐glycosylation, O‐OTase, PglL, post‐translational modifications

## Abstract

Protein glycosylation is now recognized as a ubiquitous process observed in all domains of life. Within bacterial species, carbohydrates can be attached to multiple residues with glycosylation of serine, threonine, or tyrosine residues via their hydroxyl side chains referred to as O‐linked glycosylation. To date, multiple bacterial enzymes have been identified that mediate O‐linked glycosylation targeting either surface or periplasmic bacterial proteins, and in the case of toxin/effectors, host proteins. Within this review, we discuss the current understanding of common bacterial O‐linked glycosylation systems and the glycoproteomic approaches which have been used to characterize these events. Focusing on O‐oligosaccharyltransferases (O‐OTases), flagellin‐specific glycosylation systems, and glycosyltransferase toxin/effectors, we discuss the characteristics of known glycosylation systems. For the general O‐linked systems mediated by the PglL oligosaccharyltransferases, we outline the key considerations when assessing glycosylation events across the *Burkholderia, Neisseria*, and *Acinetobacter* genera. In addition, we highlight the technologies and advancements in glycoproteomic techniques, as well as the bioinformatic tools that now facilitate high throughput studies of O‐linked glycosylation within bacterial species. Combined, this review outlines our current understanding of O‐linked glycosylation within well characterized Gram‐negative genera and the current technologies available for exploring bacterial O‐glycoproteomes.

## 1 Introduction

Polysaccharide‐rich structures are a hallmark of the prokaryotic cell envelope, playing critical roles in maintaining cellular integrity and facilitating interactions with the extracellular environment [1, 2, 3]. Within prokaryotes, carbohydrates serve as integral components of structural polymers, such as peptidoglycan, lipopolysaccharide, and teichoic acid, which are required to maintain cellular rigidity as well as determine cell shape [1, 2, 3]. However, beyond their role as surface components, carbohydrates are increasingly recognized as an important mechanism for reshaping the proteome through the covalent attachment of carbohydrates to proteins, resulting in a diverse class of post‐translational modifications known as protein glycosylation events [4]. The importance of glycosylation for modulating protein function, structure, and cellular interactions is emphasized by its universal observation across all domains of life [5, 6]. While studies first identifying archaeal and bacterial glycosylation were published nearly 60 years ago [7, 8], it was not until the 2000s that glycosylation began to be recognized as a more widespread phenomenon within bacterial systems [9, 10, 11, 12, 13]. Over the past two decades, significant advancements have been made in understanding the structural and biosynthetic pathways associated with bacterial glycosylation, enhancing our knowledge of their roles in microbial physiology and host interactions.

To date, a diverse array of bacterial glycosylation systems has been identified [5, 6, 14, 15, 16], differing in the protein classes they target, the amino acid residues that can be modified, and the carbohydrates that can be mobilized. Many of these features have been extensively reviewed by others [5, 6, 14, 15, 16, 17] with the focus of this review being on our understanding of the O‐linked glycoproteomes within Gram‐negative species. O‐linked glycosylation is defined as the attachment of either monosaccharaides or polysaccharides to hydroxy containing amino acids including serine, threonine, and to a lesser extent tyrosine. In contrast, N‐linked glycosylation is characterized by the attachment of glycans to asparagine residues with this form of glycosylation observed across members of the *Campylobacter* genus [10, 18, 19, 20] as well as within species such as *Haemophilus influenzae* [21] and *Actinobacillus pleuropneumoniae* [22], yet these systems will not be discussed in this review. Across known bacterial species, several discrete O‐linked glycosylation systems have been identified, each linked to various processes, including host colonization, motility, pathogenicity, and immune modulation [23, 24, 25, 26, 27, 28]. Within bacteria, O‐linked glycosylation is thought to regulate key protein properties, such as stability, folding, proteolytic cleavage, and solubility, which is hypothesized to contribute to bacterial adaptability and survival in diverse host environments [29, 30, 31, 32, 33, 34]. Therefore, understanding O‐linked glycosylation is critical for providing mechanistic insights into bacterial proteins, with mass spectrometry now serving as an indispensable tool for studying these events. The significant expansion in the identification of bacterial glycosylation over the last 20 years is largely due to the tremendous improvements in mass spectrometry capacities [35] and sophistication of glycoproteomic techniques [36]. These advancements have not only facilitated the characterization of bacterial glycosylation events but also expanded the number of teams now able to study these modifications.

In this review, we summarize our current understanding of common bacterial O‐linked glycosylation systems. Cataloging the trends and shared properties of different O‐linked glycotransferases/oligosaccharyltransferases, we compare and contrast the unique characteristics that set them apart, including their protein preferences. Focusing on the general O‐linked glycosylation systems of the *Burkholderia*, *Neisseria*, and *Acinetobacter* genera, we discuss the field's current understanding of the O‐glycoproteomes of these organisms and the increasing recognition that, while mechanistically similar, these glycosylation systems have distinct characteristics. Finally, we provide an overview of the current tools and approaches for studying bacterial glycoproteomics, providing examples of how these tools have been used to investigate O‐linked glycosylation events.

### 1.1 Mechanisms of O‐Linked Glycosylation

**FIGURE 1:**
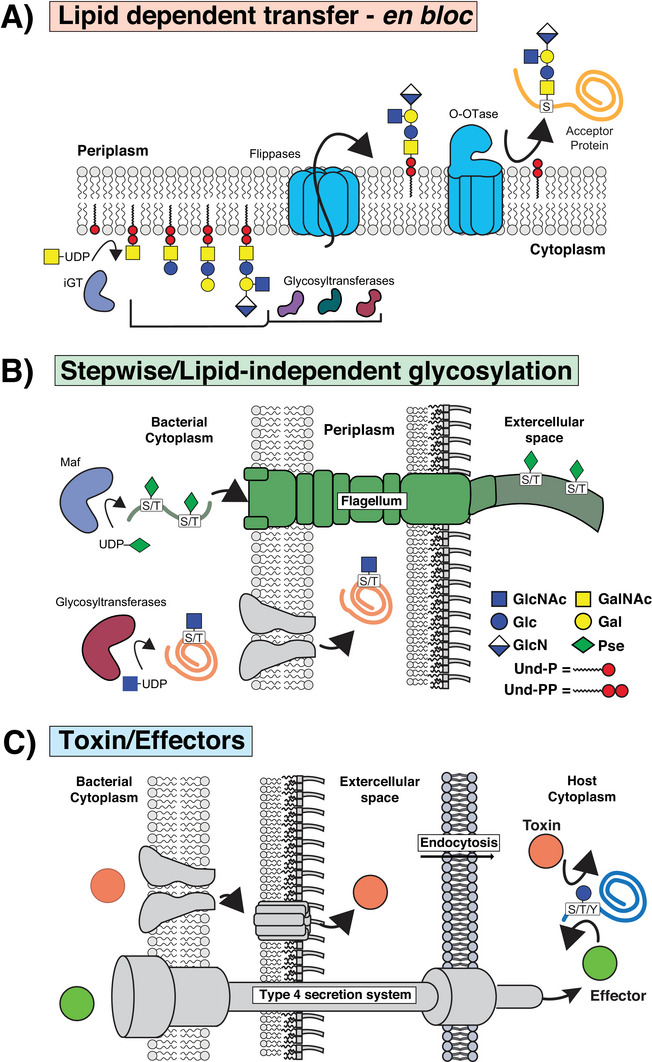
Common classes of bacterial O‐linked glycosylation systems. Bacterial biosynthetic pathways generate a diverse array of UDP sugar precursors that are utilized by glycosyltransferases for both *en bloc* (lipid‐dependent transfer) and stepwise (lipid‐independent) glycosylation pathways. (A) For *en bloc*/lipid‐dependent systems, an initiating glycosyltransferase (iGT) begins glycan biosynthesis by forming a lipid‐linked oligosaccharide (LLO) on the cytoplasmic side of the inner membrane, enabling glycan elongation by glycosyltransferases. Upon completion, the glycan is flipped into the periplasmic space by the action of a flippase, allowing transfer of the glycan to protein substrates by O‐OTases. (B) For stepwise glycosylation/lipid‐independent glycosylation, nucleotide‐activated sugars are transferred directly to flagellin subunits within the cytoplasm by Maf proteins, with the resulting glycoprotein subsequently utilized for flagellum construction. (C) Glycosyltransferase toxins and effectors encoded by pathogens are transported to host cells via secretion or translocation, enabling these proteins to glycosylate host cell proteins such as GTP‐binding proteins, thereby preventing downstream signaling pathways or protein function within eukaryotic cells.

Beyond the amino acids targeted for modification, glycosylation systems can be broadly classified based on the mechanisms by which sugars are transferred to proteins. Within this taxonomic organization, glycosylation systems can be defined as either (**I**) “*en bloc*,” also known as lipid‐dependent glycosylation systems or (**II**) stepwise, also known as lipid‐independent glycosylation systems (Figure 1) [10, 37]. Within lipid‐dependent systems, glycan construction mirrors the widely distributed bacterial Wzx/Wzy‐dependent polysaccharide biosynthesis pathways [2, 38], wherein the glycans utilized for protein glycosylation are synthesized on lipid carriers by the sequential actions of cytoplasmic glycosyltransferases [37]. Within these systems, the initiation of glycan biosynthesis on lipid carriers by initiating glycotransferases (iGST, Figure 1) acts as a scaffold for polysaccharide unit elongation by the non‐templated activities of glycosyltransferases [39]. Upon completion of glycan elongation, the resulting lipid‐linked oligosaccharides (LLOs) are translocated into the periplasmic space by translocases known as flippases [39, 40]. Once within the periplasmic space, provided the glycan of the LLO possesses the required structural features to allow transfer (vide infra, Table 1), glycosylation of compatible acceptor proteins occurs via the *en bloc* transfer of glycan units by the action of periplasmic O‐oligosaccharyltransferases [14, 41, 42]. While extensive diversity exists within O‐oligosaccharyltransferases [43], these enzymes are collectively referred to as “O‐OTases” [44] with several classes recognized to date including TfpO/PilO of *Pseudomonas aeruginosa* [45], PglL of *Ralstonia solanacearum* [46], and PglS of *Acinetobacter baylyi* [47]. In contrast to *en bloc* glycosylation, stepwise/lipid‐independent glycosylation involves the direct, sequential transfer of monosaccharides to proteins within the cytoplasm by glycosyltransferases (Figure 1) [48, 49]. Examples of lipid‐independent glycosylation systems include the flagella glycosylation systems of the pathogens *Campylobacter jejuni* [50], *Campylobacter coli* [51], and *Helicobacter pylori* [24, 52, 53]; as well as several classes of bacterial glycosyltransferase toxins [54]. Despite the functional similarity within lipid‐independent glycosylation systems, most of the known systems correspond to unique enzyme families that possess only broad sequence similarity, supporting the convergent evolution of multiple enzyme families that perform glycosylation [55].

**TABLE 1:** O‐OTase enzyme family properties.

O‐OTase	TfpO/PilO	TfpM	PglS	PglL
**Glycosylation sequon**	C‐terminal serine	C‐terminal serine or threonine	Internal serine or threonine	No defined sequon but preference for highly disordered regions rich in alanine (also P and E/D adjacent to serine in *Acinetobacter*
**Glycotag motif**	TAWKPNYAPANAPK **S**		CTGVTQIASGA **S** AATTNVASAQC (or terminal S)	WPAAA **S** AP
**Sugar at the reducing end of the substrate transferred**	Acetamido group at the C2	Acetamido group at the C2 /Galactose / Glucose	Acetamido group at the C2 / Galactose / Glucose	Acetamido group at the C2 and Galactose
**Glycan size tolerance**	Limited (< 2 kDa)	Variable	Broad (> 10 kDa)	Variable
**Target specificity**	Specific	Specific	Specific	Broad
**Native substrate**	PilA	Type IV pilin‐like proteins	ComP	Multiple targets
**Functionality observed within**	Acinetobacter, Pseudomonas	Acinetobacter, Moraxella	Acinetobacter	Neisseria, Vibrio, Acinetobacter, Burkholderia, Francisella, Ralstonia
**Substrate genetic location**	O‐Tase closely genetically linked	O‐Tase closely genetically linked	O‐Tase closely genetically linked	Not genetically linked yet can target genes in proximity to O‐Tase
**References**	[45, 47]	[42]	[47, 75]	[12, 46, 174, 175]

### 1.2 O‐Oligosaccharyltransferase Glycosylation Systems

**FIGURE 2:**
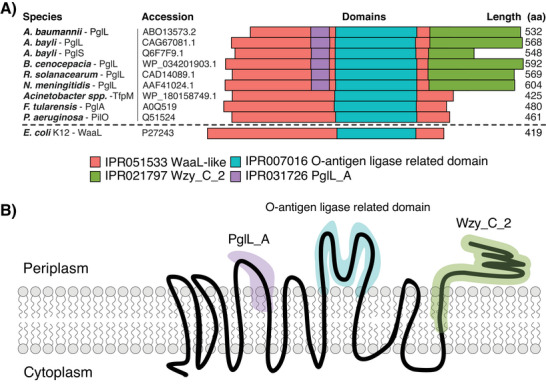
The domain structure and topology of O‐OTases. (A) InterProScan domain analysis reveals the conservation of domain structures despite low sequence similarities across known O‐OTases with all O‐OTases possessing WaaL‐like (IPR051533) and the O‐antigen ligase‐related domain (IPR007016) which are also conserved within WaaL. Within the PglL and PglS O‐OTase classes, PglL_A (IPR031726) and Wzy_C_2 (IPR021797) domains are also conserved yet these domains are absent in known TfpO / TfpM O‐OTases. (B) Topological modeling of the *N. meningitidis* PglL O‐OTase reveals that the domains PglL_A (IPR031726), O‐antigen ligase‐related domain (IPR007016), and Wzy_C_2 (IPR021797) are all predicted to be periplasmic facing supporting their role in facilitating glycosylation.

The O‐OTases are widespread across Gram‐negative species [42, 43], serving as a common mechanism for enabling periplasmic glycosylation (Figure 1). As noted above, multiple classes of O‐OTases have been identified, with these systems either displaying a preference for glycosylating a single substrate or targeting multiple proteins (Table 1). Three discrete protein specific O‐OTases have been identified to date including the pilin‐specific O‐OTases TfpO/PilO [45, 47] and TfpM [42], which target the carboxy termini amino acid of pilin substrates [42, 56], and PglS which has been shown to glycosylate internal regions of type IV pilin‐like ComP proteins [47, 57, 58]. In contrast, the PglL class of O‐OTases has broader targeting specificity and glycosylates multiple proteins within different bacterial species [14, 47]. Bioinformatically, different classes of O‐OTases share relatively low amino acid sequence similarity yet all known members possess both a central O‐antigen ligase related domain (IPR007016) and a WaaL‐like (IPR051533) domain (Figure 2A) [43]. As both the IPR007016 and IPR051533 domains are present in WaaL O‐antigen ligases required for lipopolysaccharide (LPS) biosynthesis, this limits the use of these domains alone to assign putative O‐OTases. In the case of PglL and PglS O‐OTases, the co‐occurrence of these domains with the PglL_A (IPR031726) and Wzy_C_2 (IPR021797) domains has been highlighted to allow the detection of these proteins [41, 43] yet these domains appear less conserved within the TfpO and TfpM classes of O‐OTases (Figure 2A). While the precise role of these domains is unclear, topological predictions suggest the IPR031726, IPR021797, and IPR007016 domains are periplasmic facing, supporting their involvement with mediating the glycosylation process (Figure 2B) [43, 59].

For several O‐OTases including TfpO/PilO, TfpM, and PglS, the cognate substrates of these O‐OTases are often encoded in close proximity to their respective targets [42, 45, 47]. However, this tight genetic linkage is not widely observed within the PglL O‐OTases, consistent with their function as “general” O‐OTases responsible for the glycosylation of diverse periplasmic targets. An important nuance within the O‐OTase systems is that while different classes exist, similar substrates can still be targeted by each class of O‐OTase, for instance, pilin/pilin‐like proteins can be glycosylated by general O‐OTases as well as TfpO, TfpM, or PglS enzymes. This is best illustrated by the major pilin subunit (PilE) of *Neisseria gonorrhoeae* [60] and *Neisseria meningitidis* [61] which are both glycosylated by PglL O‐OTases yet these O‐OTases also glycosylate multiple extracytoplasmic proteins within these species [12, 13, 62, 63]. A similar observation has been noted within *Francisella tularensis* where the O‐OTase PglA glycosylates Pilin [64] as well as additional endogenous substrates [65]. In addition to the differences in protein repertoires between O‐OTase classes, differences can also exist between O‐OTase orthologs within the same class [62, 63]. While this observation was first noted within studies assessing the functionality of different PglL variants using reporter proteins [62, 66], these differences have now been observed at a proteomic scale [62, 63] supporting that different PglL variants possess discrete protein substrate ranges. These trends demonstrate that glycosylation of a specific substrate alone does not provide insights into the class of O‐OTase associated with these events, nor does the observation that O‐OTases of the same class ensure similar proteins will be glycosylated within different bacterial species.

Across the known O‐OTase glycosylation systems, variable patterns of glycan conservation within protein O‐linked glycosylation are observed, ranging from extensive variability across isolates noted in *Acinetobacter baumannii* [67, 68] to the nearly genus‐wide conservation as seen in the *Burkholderia* genus [69, 70, 71, 72]. This diversity suggests that generalizable trends in glycan selection may be limited for O‐OTase glycosylation systems. However, insights into the specific requirements for glycan mobilization have emerged with the reducing sugar now known to be important in governing glycan compatibility within several O‐OTase classes (Table 1). Within the TfpO/PilO [73] and PglL [44, 74] classes, glycan mobilization typically requires the reducing sugar to possess an acetamido group at the C2 position with galactose also shown to be transferrable by the *N. meningitidis* PglL [74]. In contrast, the PglS [75] and TfpM [42] classes of O‐OTases are capable of transferring diverse arrays of carbohydrates including glycans containing glucose as the reducing sugar, making them attractive tools for glycoengineering purposes [14, 76]. Beyond the specificity for the reducing sugar highlighted for the TfpO/PilO [73] and PglL [44, 74] O‐OTases, the composition of glycans beyond the first sugar appears to have little impact on glycosylation aside from reported differences in the size of the glycans that can be effectively transferred. Across O‐OTase classes, several O‐OTase variants possess the ability to transfer large polysaccharides generated by the polymerization of glycan units, leading to the addition of glycan chains > 5 kDa to protein substrates [42, 44, 66, 77]. These enzymes are thus of considerable interest for glycoengineering efforts [14, 76]. However, these differences appear to be characteristic of different O‐OTase variants, rather than classes. For example, while the *N. meningitidis* PglL enzyme is able to transfer glycans of varying lengths [44, 74], the PglL enzyme of *A. baumannii* demonstrates a preference for glycan units < 2 kDa in size [67, 68]. Similarly, within the TfpM class of O‐OTases, different orthologs possess varying capacities to transfer highly polymerized glycans [42]. These features suggest that while diverse glycans can be utilized by O‐OTases, these enzymes exhibit preferences for specific glycans and glycan lengths, which need to be assessed on a case‐by‐case basis.

### 1.3 O‐linked Flagellin Glycosylation

Flagellin glycosylation represents a common class of O‐linked glycosylation events observed across Gram‐negative and Gram‐positive bacterial species [78, 79]. Characterized by the attachment of carbohydrates to flagellin (Figure 1), these glycosylation events play a pivotal role in modulating the assembly of flagellar filaments [80, 81], bacterial motility [24, 82, 83, 84], and modulating the host response [28, 85]. A key feature of flagellin glycosylation systems are the broad diversity in carbohydrate composition, ranging from the attachment of single monosaccharides [24, 28, 86, 87] to elongated glycans [88, 89] and even glycans containing non‐carbohydrate modification such as phosphonate moieties [27, 90, 91]. Even within flagellin glycosylation events associated with the attachment of single monosaccharides, extensive chemical diversity is observed across species, as previously summarized by Merino and Tomás [78], as well as within species in the form of glycan microheterogeneity, which has been extensively highlighted within *C. jejuni* [86, 92, 93]. This chemical diversity supports the existence of mechanistically diverse flagellin glycosylation pathways; however, the underlying enzymology associated with these glycosylation systems is still poorly defined [78, 79]. Despite our incomplete understanding of flagellin glycosylation, these events appear to occur exclusively within the cytoplasm prior to flagellin exportation with many of these glycosylation systems also closely genetically linked to their cognate flagellin substrates [78]. Among the known flagellin glycosylation systems, several glycosyltransferases are assigned as motility accessory factors (Mafs) due to their requirement for motility [24, 48, 50, 94, 95, 96]. It has now been observed that differences in substrate recognition also exist across identified Maf glycosyltransferases supporting the evolution of distinct flagellin‐binding modes within Mafs [48]. Across the identified Mafs, no known glycosylation sequon has been recognized, suggesting substrate features beyond amino acid motifs such as patterns in hydrophobicity may guide glycosylation to specific sites [24, 86, 97]. While it has been demonstrated that flagellin glycosyltransferases can target heterologously expressed flagellin monomers [98], different flagellin glycosyltransferases have been shown to possess discrete targeting preferences [48]. While these findings support the existence of differences in the flexibility of flagellin glycosyltransferases for varying targets, highly promiscuous flagellin glycosyltransferases capable of utilizing diverse substrates and compatible glycan donors have also been identified [99], highlighting their potential use in glycoengineering applications [100]. Due to the diversity within these glycosylation systems, this necessitates the use of glycoproteomic analysis to define the glycans, as well as the sites targeted by unique Mafs.

### 1.4 O‐Glycosyltransferase Toxins/Effectors

Beyond O‐glycosylation's role in bacterial physiology, several pathogenic bacterial species use glycosylation as a mechanism to subvert the host during infection, via the use of glycosyltransferase toxins and effectors (Figure 1) [54]. To date, O‐linked glycosylation‐mediated glycosyltransferase toxins and effectors have been identified within a range of pathogens including *Clostridia*, *Photorhabdus*, *Yersinia*, and *Legionella* species [54, 101], including several recently identified glycosyltransferase variants [102]. The first of these toxins to be identified was in the gastrointestinal pathogen *Clostridium difficile*, where two glucosyltransferase toxins, TcdA and TcdB, are responsible for glycosylation of the small GTP‐binding proteins Rho, Rac, and Cdc42 at threonine residues with the monosaccharide glucose [103, 104]. Glycosylation of these proteins has been shown to block the binding of GTP enhancing damage to the gut mucosa during *C. difficile* infection [103, 104]. Across the Clostridial genus, additional homologs of the prototypic TcdA/TcdB toxins have now been identified, including the *Clostridium sordellii* lethal toxin (TscL), the *C. sordellii* hemorrhagic toxin (TcsH), the *Clostridium novyi* alpha toxin (Tcn‐alpha), and the *Clostridium perfringens* large cytotoxin (TpeL) with these toxins collectively known as the Clostridial glucosylating cytotoxin family [105, 106]. Similar to Clostridial toxins the entomopathogen *Photorhabdus asymbiotica* utilizes a glycosyltransferase toxin (PaTox) that mediates O‐GlcNAcylation of tyrosine residues within the GTP‐binding protein Rho, blocking GTPase activity [107]. Optimal glycosyltransferase activities of the Clostridial toxins and PaTox require the binding of the host chaperonin TRiC/CCT system, which is thought to limit the activity of the toxins until internalized within host cells [108]. Across *Yersinia* species several glycosyltransferases have been identified in *Yersinia mollaretii*, *Yersinia enterocolitica*, and *Yersinia kristensenii*, with these toxins sharing sequence similarity to TcbB yet targeting different host substrates [102, 109]. Notably, in the case of *Y. mollaretii*, these glycosyltransferases target Rab5 and Rab31 at threonine residues for GlcNAcylation [109] while in the case of *Y. enterocolitica* and *Y. kristensenii* these glycosyltransferases target Rho, Rac, and Cdc42 at tyrosine residues for glucosylation [102]. Another notable grouping of O‐Glycosyltransferases are the *Legionella pneumophila* glucosyltransferase effectors [101, 110] of which at least five O‐linked glucosyltransferases have been identified (Lgt1‐3, SetA, and LtpM) [111, 112, 113, 114]. These glucosyltransferases are translocated directly into host cells via the actions of the Dot/Icm Type IV secretion system, where they modify host targets [115]. For example, Lgt1 is responsible for the glycosylation of the elongation factor 1A on serine residues [115], while SetA is responsible for glycosylation of threonine residues within Rab1a, Snx1, and Snx3 [116]. These examples highlight that bacterial systems are not limited to glycosylating their own proteins and several different systems exist to modify host proteins, modulating the course of infections. The observation that even glycosyltransferases with sequence similarity can target different residues and install different monosaccharides underscores the need for careful assessment of these glycosylation events.

## 2 Current Understanding of PglL O‐Linked Glycoproteomes

Across PglL O‐OTases, our understanding of their substrates and the nuances within these systems has dramatically improved over the last decade. Of the known PglL systems, the Burkholderia, *Acinetobacter*, and *Neisseria* genera possess conserved systems that are arguably the most well‐understood. In these genera, glycoproteomics has played a critical role in both the characterization of these glycosylation pathways and the elucidation of glycoprotein substrates. In the following sections, we summarize the key attributes associated with the glycoproteomes of these genera and discuss how our understanding of these systems can help guide the assumptions made during the analysis of these glycoproteomes.

### 2.1 Burkholderia

The *Burkholderia* genus possesses a highly conserved PglL‐mediated O‐linked glycosylation system, with general O‐linked glycosylation confirmed within all species assessed to date [71, 72, 117, 118]. The first evidence for PglL‐mediated glycosylation in this genus was demonstrated by the Feldman lab in 2012, where they showed the capacity of *Burkholderia thailandensis* PglL to glycosylate multiple proteins under heterologous expression conditions [66]. In 2014, PglL of *Burkholderia cenocepacia* (BCAL0960) was demonstrated to mediate O‐linked glycosylation of native substrates with a trisaccharide corresponding to Hex‐HexNAc‐HexNAc (observed mass 568.21 Da) or a modified trisaccharide carrying a 100.016 Da modification on the terminal hexose residue (observed mass 668.23 Da), assumed but not confirmed to be succinylation [118]. This trisaccharide has now been identified as β‐Gal‐(1,3)‐α‐GalNAc‐(1,3)‐β‐GalNAc and is encoded by a highly conserved five‐gene cluster, known as the O‐glycosylation cluster (OGC) [72] with putative succinylation also observed across all *Burkholderia* species [71]. A unique feature of *Burkholderia* glycosylation is the limited glycan heterogeneity observed beyond the two reported glycoforms in the majority of species examined, with alternative glycans only noted in *Burkholderia pseudomallei* [71]. This limited heterogeneity appears to be unique to the *Burkholderia* genus, as even closely related beta‐proteobacteria such as *R. solanacearum* exhibit extensive O‐linked protein glycosylation glycan heterogeneity [46]. At the glycoprotein level, *Burkholderia* species appear to possess relatively large glycoproteomes, with at least 141 proteins subjected to PglL‐mediated glycosylation in *B. cenocepacia* [69, 70, 71, 119]. Across the *Burkholderia* genus glycoproteins appear to be conserved in sequence and glycosylation status across species [69]. At the glycosylation site level, while initial studies highlighted the restriction of glycosylation to disordered regions within periplasmic proteins [118], site localization studies have now demonstrated a near‐exclusive preference for glycosylation on serine residues within *Burkholderia* species with threonine glycosylation only observed upon overexpression of PglL [69]. From these findings, clear guidelines can be established for assumptions when assessing *Burkholderia* glycoproteomes, including the presence of limited glycan diversity and glycosylation being restricted to serine residues in disordered regions of periplasmic proteins. From comparative studies, the observation that glycosylation within one species provides predictive insights into glycosylation in other members of the genus [69] supports the conservation of glycosylation on shared proteins across *Burkholderia* species.

### 2.2 Neisseria

*Neisseria* species exhibit extensive variability in their glycoproteomes characterized by significant intra‐ and inter‐strain differences in the glycan compositions used for protein glycosylation [120, 121, 122, 123, 124]. Within this genus, glycan heterogeneity is mediated by the following three key factors: polymorphic glycosyltransferase content, phase variable expression of glycosyltransferases within strains, and O‐acetylation of glycans. Allelic variations in glycan biosynthesis enzymes have been observed in multiple genes of the *p*rotein *g*lycosylation *l*oci, referred to as the *pgl*. For example, differences in the phosphoglycosyltransferase alleles PglB/B2 have been demonstrated to result in the incorporation of different reducing sugars, switching from N,N′‐Diacetylbacillosamine (diNAcBac) to Glyceramido‐acetamido‐trideoxyhexose [125]. Similarly, changes in the alleles of the PglH/H2 glycosyltransferases have been shown to switch the incorporation of monosaccharides within glycans from glucose to N‐Acetylglucosamine, contributing to glycan diversity [122, 126]. Importantly, the expression of additional glycosyltransferases can further expand the glycan repertoire in *Neisseria* species as observed for the expression of PglG which is variably observed within *N. meningitidis* and *N. lactamica* species [127]. Beyond allele differences and differences in *pgl* content, many *pgl* genes undergo phase variation, leading to altered expression and further augmenting the diversity of glycans observed on glycoproteins [128, 129]. In addition to variations in *pgl* content and expression, individual carbohydrates within the glycans of *Neisseria* glycoproteins can also be subjected to varying levels of acetylation by PglI [122, 123]. Sharing the domain Pfam_PF01757 observed in acetyltransferases required for LPS acetylation, PglI can mediate the O‐acetylation of galactose and glucose residues within glycan structures expanding the observed glycan diversity [122, 123]. Due to the isobaric nature of O‐acetylation and N‐acetylation, this makes the assignment of even monosaccharide compositions within *Neisserial* glycans based on mass alone potentially ambiguous within this genus [123].

In terms of the glycoproteome of *Neisseria* species, while Pilin of *N. gonorrhoeae* and *N meningitidis* were the first glycoproteins identified [60, 61], recent surveys suggest that at least 52 glycoproteins exist across these two species [130]. However, there is a paucity of insights into the glycoproteomes of other *Neisseria* species, with only limited glycoproteomic analyses conducted to date [62, 131]. While many glycoproteins appear shared between *Neisseria* species [130], recent assessments of different *Neisseria* PglL variants have demonstrated that the glycoproteome between species may differ significantly due to PglL variants possessing discrete targeting ranges [62, 63]. Consequently, this supports that even if proteins possess high sequence identity between strains and are known to be glycosylated in one *Neisseria* species, it cannot be assumed that they will be glycosylated in another. Additionally, in *Neisseria* species, other modifications can co‐occur on glycoproteins, such as the addition of phosphocholine and phosphoethanolamine [132, 133]. These modifications are known to occur in close proximity to glycosylation events [132, 133], or even on sites known to be glycosylated [133]. At the glycosylation site level, similar to other PglL systems, glycosylation in *Neisseria* occurs within disordered regions, with all localized sites to date occurring on serine residues [12, 121]. Based on these findings, when assessing *Neisseria* glycosylation, minimal assumptions should be made regarding putative glycan compositions. While glycans observed within species may share similarities with previously reported glycans microheterogeneity is likely to also be observed. Although prior observations of glycosylation within a conserved protein may suggest its potential glycosylation in a different species, the discrete targeting ranges of PglL variants ultimately determine the glycosylation status of a given protein. Finally, due to the potential co‐occurrence of phosphocholine and phosphoethanolamine, these modifications should also be considered when analyzing *Neisseria* glycoproteins.

### 2.3 Acinetobacter

*Acinetobacter* O‐linked glycosylation was first identified within *A. baumannii* where PglL (A1S_3176) was demonstrated to mediate O‐glycosylation [134]. Sequence analysis and functional studies have now shown that O‐glycosylation is conserved and functional across several *Acinetobacter* species indicating it is a ubiquitous process within this genus [47, 67]. Within *Acinetobacter* species, the *O‐*linked glycans used for protein glycosylation are derived from capsule monomers (referred to as K‐units) produced by the highly variable K loci [135]. The genetic variability between isolates in the K loci leads to the generation of diverse K‐units differing in glycosidic linkages, length of the glycan, and monosaccharide composition [67, 136, 137]. Notably, several rare monosaccharides are utilized within these glycans across the different K‐loci, including non‐ulosonic sugars such as Pseudaminic (Pse), and Legionaminic acids [138, 139, 140, 141]. Heterogeneity within K‐unit glycan structures, including variations in acetylation and methylation levels, have been previously noted, with the degree of this heterogeneity appearing to be strain‐specific [67, 134]. From glycoproteomic assessments, O‐linked glycosylation predominately involves the addition of unpolymerized or minimally polymerized K‐units fewer than 10 sugar monomers in length by PglL to proteins [67, 142]. To date, 33 unique glycoproteins and 42 glycosylation sites have been confirmed across various *A. baumannii* strains [67, 68] with *A. baumannii* PglL shown to be a serine‐specific *O‐*Tase [68]. These glycosylation events occur within highly disordered protein regions rich in alanine and proline, with glycosylation taking place near negatively charged aspartic and glutamic acid residues [68]. Analysis of *A. baumannii* glycosylation site conservation suggests identified sites appear conserved across strains [68] yet it is currently unclear how conserved the *Acinetobacter* glycoproteome is outside of *A. baumannii* beyond preliminary analysis of *A*. *baylyi* [47]. While PglL is highly conserved in the *Acinetobacter* genus, strains can also possess additional O‐OTases classes with distinct targeting specificities, glycan tolerances, and preferences for glycan length (Table 1) [42, 47]. The presence of multiple O‐OTase classes within the same *Acinetobacter* species can result in O‐linked glycoproteins that possess identical glycans but are mediated by different O‐OTases [47]. This variability limits one's ability to reliably assign a specific O‐OTase class solely based on the observed glycans. From these findings when assessing *Acinetobacter* glycosylation, it is suggested unless the K‐loci and K‐unit structure are known assumptions about the composition of the glycan should be minimal. Similarly, the observation of glycoproteins containing the same glycan does not alone support that the same O‐OTase is responsible for glycosylation. Finally, as PglL‐mediated glycosylation demonstrates a preference for serine residues, the observation of glycosylation on threonine residues or the carboxyl‐terminal amino acid of a protein may indicate the presence of an alternative O‐OTase class.

## 3 Glycoproteomics

**FIGURE 3:**
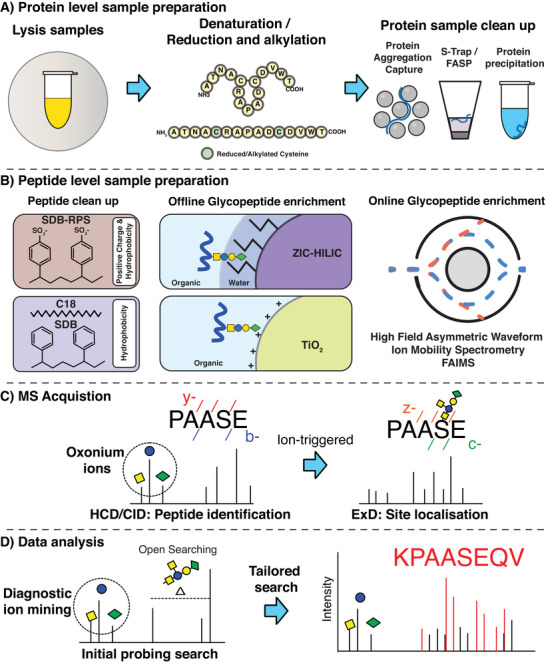
Sample preparation steps for bacterial glycoproteomics. (A) For optimal sample preparation, bacterial glycoproteins must be extracted with detergent‐based solubilization offering an effective means to prepare samples for high‐throughput glycoproteomics studies from whole cell samples. Once isolated, protein cleanup approaches such as Protein Aggregation Capture, S‐Trap, or acetone precipitation can be used to remove detergents and other potential contaminants. Prepared protein samples can then be digested with a range of enzyme(s) to generate glycopeptides for subsequent analysis. (B) To enhance the study of glycosylation, proteome samples can be cleaned up to enable the enrichment of glycopeptides. This can be done using offline methods such as ZIC‐HILIC or TiO_2_ chromatography prior to MS acquisition, or via online methods such as FAIMS fractionation. (C) During MS acquisition, ion‐triggering methods where the presence of specific fragment ions (diagnostic ions) in collision‐induced dissociation (CID) scans triggers additional MS2 scans (e.g., ExD) can be used to provide localization information as well as complementary data on the glycopeptide identity. (D) Data analysis of bacterial glycopeptides can be performed using computational strategies, including open searching and ion mining‐based approaches, to determine glycan masses and diagnostic ions specific to the glycosylation patterns observed within a specific bacterial isolate. This information can be used to tailor MS acquisition for ion‐triggering experiments and to create customized searches within search engines, improving the identification of glycopeptides.

The central goal of glycoproteomics is to characterize glycosylation by determining the location of glycosylation sites and the attached glycan compositions using proteomic and mass spectrometry techniques. In bacterial glycosylation, the diversity in the machinery used to install glycosylation and the resulting glycan diversity (vide supra) creates a situation where many of the assumptions widely implemented in eukaryotic glycosylation [36] do not hold true in bacterial systems. For example, due to the diverse array of unique monosaccharides incorporated into bacterial glycans [17], this limits the compatibility of many commercial enzymes used for eukaryotic glycoproteomics, such as glycosidases, glycoproteases such as mucinases, and deglycosylating enzymes [36, 143]. Due to the unique chemical diversity of glycoproteomes, this has notable impacts on several areas of glycoproteomics workflows, including sample preparation, enrichment, data collection, and data analysis (Figure 3). In this section, we outline considerations when undertaking glycoproteomic analysis and current best practices within the field to address the challenges associated with these systems.

### 3.1 Sample Preparation

Sample preparation is crucial for robust quantitative analysis and for maximizing the coverage of glycoproteins observed. A key consideration when assessing bacterial glycoproteomes is how to achieve optimal extraction of glycoproteins, as many can be integral membrane proteins, making them challenging to solubilize using mild lysis buffers [144]. While protein level fractionation through ultracentrifugation has been used to improve access to membrane glycoproteins [134], increasingly whole‐cell‐based analysis is utilized due to its simplicity and the recognition that many glycosylation events are readily observable within these preparations [46, 70, 71, 118]. In early studies of bacterial glycoproteomes, generally mild solubilization approaches using chaotropic agents such as thiourea/urea dominated the field [46, 70, 71, 118, 134] due to their compatibility with both protein separation approaches, such as 2D gel electrophoresis, as well as in‐solution digestion protocols. A key change in recent years has been the transition away from chaotropic agents for sample preparation to the use of detergent‐based methods, such as lysis with sodium dodecyl sulfate (SDS) [62, 63, 68] or sodium deoxycholate (SDC) [117, 119] for sample preparation. The use of detergents allows both rapid denaturation by boiling of samples while also improving the recovery of membrane proteins during sample lysis [145]. The increasing use of detergent‐based solubilization approaches reflects advancements in proteomic protein cleanup techniques, including the emergence of techniques such as Protein Aggregation Capture/Single‐Pot Solid‐Phase‐Enhanced Sample Preparation (SP3), Filter‐Aided Sample Preparation (FASP), and S‐Trap‐based approaches which are all highly effective at removing detergents during sample preparation (Figure 3) [146, 147, 148]. In addition to improving the extraction of membrane proteins, these approaches, along with others such as the minimal encapsulated proteomic sample (in‐StageTip) approach [149], can minimize sample loss due to reduced sample handling. A key additional benefit of the streamlining of sample preparation is improved reproducibility, which enhances quantitative assessments of bacterial glycosylation events which are increasingly being undertaken on bacterial glycoproteomes [62, 63, 117, 119].

While the extraction of glycoproteins is essential for the analysis of bacterial glycosylation, the detection of glycoproteins can be heavily influenced by the protease/s used during digestion. Across proteomic studies, trypsin is the dominant protease used; however, this protease can be suboptimal for many bacterial glycosylation systems, which has motivated the use of alternative proteases in several studies [68, 69]. For example, within PglL O‐OTases, the preference for glycosylation sites in disordered regions results in many glycosylation sites being missed when trypsin alone is used due to the low frequency of lysine and arginine in these regions [12, 68, 69, 121]. In such systems, the use of alternative proteases or combinations of proteases can improve the detection of glycosylation events that would otherwise be undetected. The use of alternative proteases has proven useful in the study of *A. baumannii* and *B. cenocepacia* glycoproteomes, where the use of multiple proteases—including non‐specific proteases such as pepsin and thermolysin—has enabled the identification of novel glycoproteins [68, 69]. Beyond the use of non‐specific proteases, combinations of proteinases such as AspN and trypsin have also been used to enhance the localization of glycosylation events within the Flagellin of *B. cenocepacia* improving access to regions within this protein inaccessible to a single protease alone [28]. It is worth noting that non‐specific proteases, such as proteinase K, have also been used in several studies [12, 74, 87, 134], which offer some unique benefits for glycoproteomic analysis by allowing the generation of short peptide fragments which may contain only a single hydroxyl‐containing amino acid. The generation of short peptides with only a single hydroxyl‐containing amino acid can allow the precise assignment of glycosylation events, provided the peptide sequence is unique to a single region of the protein.

### 3.2 O‐Glycopeptide Enrichment

Due to the variable levels of occupancy, as well as glycan heterogeneity, at the proteome level glycopeptides are typically less abundant than non‐glycosylated peptides [150]. Therefore, the selective enrichment of glycopeptides is an ideal strategy to improve glycopeptide analysis by depleting non‐glycosylated peptides (Figure 3) [36]. Several approaches for the enrichment of bacterial glycopeptides have been demonstrated exploiting the unique chemical properties of glycopeptides, including their hydrophilicity, glycan charge, and ion‐mobility differences compared to non‐glycosylated peptides [36]. One of the most widely used methods for glycopeptide enrichment in bacterial glycoproteomics is ZwitterionIC Hydrophilic Interaction LIquid Chromatography (ZIC‐HILIC) [151, 152]. This approach exploits the hydrophilic nature of glycans, enabling the isolation of glycopeptides from less hydrophilic, non‐glycosylated peptides [151, 152]. ZIC‐HILIC enrichment has been used for O‐glycoproteomic analysis in several bacterial systems, including *Acinetobacter*, *Burkholderia*, and *Ralstonia* [46, 67, 118, 134]. While effective, the overall hydrophilicity of glycopeptides influences enrichment efficacy, with specificity and selectivity adjustable by altering buffer compositions such as ion‐pairing agents [152] or organic solvents used [153]. Charge‐based enrichment has also been utilized in bacterial glycoproteomics, leveraging the affinity of negatively charged carbohydrates to positively charged resins [68, 91, 124]. Titanium dioxide (TiO₂) chromatography, commonly used for phosphopeptide enrichment, has proven effective for enriching negatively charged sialic acid‐containing glycans observed in eukaryotic glycopeptides [154, 155]. TiO₂’s selectivity for negatively charged glycans has been exploited to enrich *Neisseria* glycopeptides containing glucuronic acid (GlcA) [124] and *A. baumannii* glycopeptides containing GlcNAc3NAcA4OAc and Pse [68]. Notably, phospho‐containing moieties within glycans can also be enriched using this approach, as demonstrated with the enrichment of flagellar glycosylation events of *C. difficile* [91]. An emerging technique for identifying bacterial glycosylation events is ion mobility‐based fractionation, such as high‐field asymmetric waveform ion mobility spectrometry (FAIMS). FAIMS separates gas‐phase ions based on size and shape [156], offering a tractable method to fractionate proteome samples and improve the detection of bacterial glycosylation [70, 157]. This approach has been used to enhance glycoproteome coverage in *Burkholderia*, revealing glycopeptides undetected by ZIC‐HILIC enrichment [70]. FAIMS has also been applied to the *N. gonorrhoeae* glycoproteome to detect variations in glycosylation due to alterations in PglL alleles, demonstrating the capability to analyze glycosylation occupancy changes [62, 63]. However, not all bacterial glycoproteomic systems benefit equally from FAIMS fractionation. For example, within studies of *A. baumannii* glycosylation it has been observed that no unique glycopeptides were identified with FAIMS compared to TiO₂ or ZIC‐HILIC enrichment approaches across several different *A. baumannii* strains [68]. This underscores the importance of tailoring enrichment approaches to the bacterial glycosylation system under investigation, as multiple strategies may yield different insights into the glycoproteome or be more efficient/effective for a given system.

### 3.3 Fragmentation of O‐Linked Glycopeptides

**FIGURE 4:**
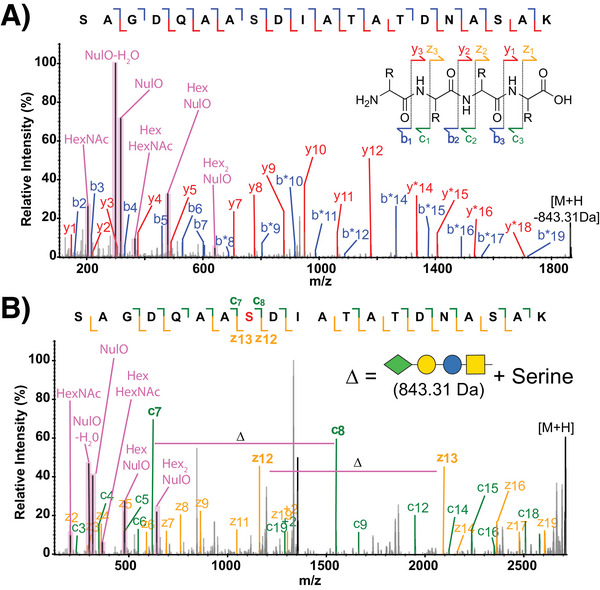
Different fragmentation approaches provide complementary glycopeptide information. Collision‐based as well as electron‐driven fragmentation approaches provide unique information for the assignment of bacterial glycopeptides. For example, consider the *A. baumannii* BAL062 glycopeptide “SAGDQAASDIATATDNASASK” glycosylated with a single 843.31 Da glycan corresponding to Pse‐Gal‐Glc‐GalNAc on serine position 8. (A) While collision‐based fragmentation provides peptide sequence information in the form of y and b ions, no localization information can be observed, with * indicating labile loss of glycan. (B) In contrast, electron‐based fragmentation results in c and z ions, allowing the localization of the glycan to position 8.

A key aspect of glycopeptide analysis is the ability to identify both the glycan component linked to a peptide and, if desired, localize these events to a specific amino acid. While several types of collision‐based fragmentation approaches are available to researchers [158], these techniques often lead to the complete or partial loss of O‐linked glycans due to their labile nature with only unmodified y/b‐ions observed. This loss prevents glycan localization on glycopeptides [159] requiring alternative fragmentation approaches, such as electron‐based (ExD) fragmentation to achieve localization. These methods retain the glycan on the resulting fragment ions, producing complementary c‐ and z‐ions that facilitate glycan localization to specific amino acid residues (Figure 3). However, ExD, especially in its most commercially prevalent form, electron transfer dissociation (ETD), can be significantly slower (10–100 ms vs. 0.1–20 ms for collision‐based approaches) [160]. Additionally, the fragmentation efficiency depends on charge density [160], making low charge density glycopeptides less suitable for this approach. To leverage the strengths of both fragmentation methods, product ion‐triggered ETD can be employed, where ETD scans are triggered only when diagnostic ions of interest are detected in the initial MS2 spectra [159, 161]. In this workflow, precursor ions are first fragmented by collision‐based fragmentation to generate product ions. Upon detection of relevant diagnostic ions, the same precursor ions undergo a second round of fragmentation using ETD or supplemental activation ETD (such as EThcD) [160]. This dual approach provides robust peptide identification through y/b‐ions while ensuring precise glycan localization through c/z‐ions. Product ion‐triggered methods often rely on common oxonium ions for triggering, such as 204.0867 *m*/*z* (HexNAc) and 366.1396 m/z (HexNAc‐Hex), enabling targeted identification of glycopeptides, as demonstrated for the analysis of *Burkholderia* glycoproteomes [69, 70, 71, 117, 119]. However, HexNAc is absent in many bacterial glycans, necessitating a more customized approach to ETD triggering with a range of glycan‐associated ions suitable for this application (Table 2). For example, several studies of *N. gonorrhoeae* glycosylation have utilized the diagnostic oxonium ions of diNAcBac, 229.1189 m/z and 211.1082 m/z, in place of the HexNAc oxonium ions to enable effective product ion‐triggering [62, 63]. Similarly, in *A. baumannii*, varying glycan compositions across different capsule types result in distinct diagnostic ions with the incorporation of tailored ion‐triggering strategies an ideal means to account for these compositional differences [68]. Beyond localization, it should be noted that using both ExD and collision‐based fragmentation approaches provide complementary information on the identity of a given bacterial glycopeptide. By collecting both types of fragmentation information offers a simple as well as orthogonal way to support the assignment of novel glycosylation events (Figure 4). Thus, the use of ETD should be considered a valuable tool to enhance confidence in glycosylation assignments, beyond just the localization information it provides.

**TABLE 2:** Common glycan‐associated diagnostic ions.

Oxonium ion	*m*/*z*	Composition
HexNAc	204.0867	C_8_H_13_O_5_N + H^+^
HexNAcHex	366.1395	C_14_H_26_O_10_N
dHexNAc	188.0917	C_8_H_13_O_4_N + H^+^
dHexNAc—H_2_O	170.0812	C_8_H_11_O_3_N + H^+^
NulO	317.1343	C_13_H_20_O_7_N_2_ + H^+^
NulOAc	359.1448	C_15_H_22_O_8_N_2_ + H^+^
NulO—H₂O	299.1238	C_13_H_18_O_6_N_2_ + H^+^
HexNAcA	218.0659	C_8_H_11_O_6_N + H^+^
diNAcBac	229.1189	C_10_H_17_O_4_N_2_ + H^+^
diNAcBac—H_2_O	211.1082	C_10_H_15_O_3_N_2_ + H^+^
HexNAcNacA	259.0926	C_10_H_15_O_6_N_2_+ H^+^
HexNAc3NacA4OAc	301.1031	C_12_H_17_O_7_N_2_+ H^+^
HexNAcOAc	246.0972	C_10_H_17_NO_7_+ H^+^

### 3.4 Computational Tools for the Identification and Visualization of Bacterial Glycosylation Events

The informatics associated with glycoproteomics analysis is continuously improving, with new tools and workflows enabling the robust identification of glycopeptides [162]. Popular search tools, including MSfragger [163], Mascot [164], and Byonic [165], have been widely used to identify bacterial glycosylation events, with comprehensive summaries of the benefits and differences between these tools available elsewhere [36, 162]. A key challenge in studying bacterial glycosylation is identifying glycosylation events given the diverse array of glycan structures, many of which are difficult to predict. Early studies often relied on manual identification of glycan structures [46, 47, 67, 118, 134, 135], which likely resulted in only a subset of glycopeptides within samples being reported due to the complexity of assigning all glycoforms or unique glycopeptides. An alternative to manual identification is the use of open‐search‐based approaches (Figure 3), which allow unknown glycan compositions to be discovered based on observed mass shifts (Δmass) between the observed modified mass and the expected unmodified mass of the peptides [71]. This approach has been invaluable in bacterial glycosylation studies, helping to identify glycan masses in systems where glycan structures are often unknown, such as O‐linked glycosylation associated with O‐OTases [42, 63, 68, 117] and glycosylation systems within the *Bacteroidota* phylum [166, 167, 168]. Using open searching in combination with strategies to identify common repeating diagnostic ions [169, 170], which typically correspond to glycan‐associated ions, allows glycan ions to be integrated into searches improving the scoring and identification of bacterial glycopeptides [171]. Critically, when assigning novel glycosylation events, visualizing the resulting fragmentation patterns and ensuring the observed ion current within a given spectrum matches the assignment provides further confidence in the assignments. While manual assignment was traditionally used to achieve this [46, 47, 67, 118, 134, 135], several tools are now available to aid in the annotation of spectra which greatly improves the capacity for non‐experts to interrogate spectra [172, 173]. Even experts are strongly encouraged to utilize these computational tools to aid in glycopeptide assignment and to ensure accurate visualization of glycosylation events.

## 4 Conclusion

The study of O‐linked glycosylation continues to mature, with an increasing number of glycosylation systems now recognized across bacterial species. While the field of bacterial O‐linked glycoproteomics was initially hampered by the lack of glycan conservation observed in eukaryotic systems, our growing understanding of bacterial glycosylation has demonstrated that glycan and substrate diversity are integral components of many, though not all, bacterial systems. By recognizing diversity not as a limitation but as a hallmark of bacterial glycosylation this has established a framework for studying these systems, wherein minimal assumptions are made regarding glycan composition, substrate ranges, and sites of localization. This framework acknowledges that despite mechanistic similarities, each glycosylation system is unique, allowing for an agnostic assessment of glycosylation within bacterial species. To achieve this agnostic assessment, tools developed for eukaryotic glycosylation provide a useful starting point [36] for investigating bacterial glycosylation events, yet they are often insufficient for analyzing the diverse glycosylation patterns found in bacteria. The emergence of tailored approaches to characterize bacterial glycoproteomes now bridges this technical gap with current generations of MS instrumentation [35] offering researchers far greater flexibility and versatility in collecting glycoproteomic data compared to earlier generations of instruments. Additionally, as bioinformatics tools continue to evolve [162], streamlined platforms for identifying diagnostic ions or fragmentation patterns unique to bacterial glycans are now accessible to teams without specific bioinformatics expertise. These advancements enhance large‐scale bacterial glycopeptide identification and localization making these studies accessible to a broad range of researchers.

The field's growing capacity to study bacterial O‐glycosylation not only improves our ability to characterize these events but also opens exciting possibilities to leverage glycosylation systems for glycoengineering applications. Multiple O‐linked glycosylation systems have been highlighted as promising tools for glycoengineering [14, 76, 100], with the increasing recognition that different enzyme variants possess different properties likely leading to the identification of new tools for glycoengineering. Expanding glycoproteomic studies to include bacterial species possessing novel glycosylation systems [42, 43] will be essential for uncovering both conserved and divergent glycosylation mechanisms which will broaden glycoengineering capacities. Ultimately, characterizing the full extent of bacterial glycosylation is not only crucial for advancing our understanding of bacterial biology but also for unlocking the therapeutic potential of bacterial‐generated glycoconjugates for use as diagnostics and vaccines.

## Conflicts of Interest

The authors declare that they have no known competing financial interest or personal relationships that could have appeared to influence this review.

## Data Availability

MS/MS data provided within Figure 4 corresponds to glycopeptide data generated in house associated with Tkalec et al. J. Proteome res 2024.
